# Cancer incidence in immigrants by geographical area of origin: data from the Veneto Tumour Registry, Northeastern Italy

**DOI:** 10.3389/fonc.2024.1372271

**Published:** 2024-05-28

**Authors:** Eliana Ferroni, Stefano Guzzinati, Alessandra Andreotti, Susanna Baracco, Maddalena Baracco, Emanuela Bovo, Eva Carpin, Antonella Dal Cin, Alessandra Greco, Annarita Fiore, Laura Memo, Daniele Monetti, Silvia Rizzato, Jessica Elisabeth Stocco, Carmen Stocco, Sara Zamberlan, Manuel Zorzi

**Affiliations:** Azienda Zero of the Veneto Region, Padua, Italy

**Keywords:** cancer, incidence, immigrant, cancer registry, country of birth

## Abstract

**Objective:**

We investigated whether there are differences in cancer incidence by geographical area of origin in North-eastern Italy.

**Methods:**

We selected all incident cases recorded in the Veneto Tumour Registry in the period 2015-2019. Subjects were classified, based on the country of birth, in six geographical areas of origin (Italy, Highly Developed Countries-HDC, Eastern Europe, Asia, Africa, South-central America). Age-standardized incidence rates and incidence rate ratio (IRR) were calculated, for all cancer sites and for colorectal, liver, breast and cervical cancer separately.

**Results:**

We recorded 159,486 all-site cancer cases; 5.2% cases occurred in subjects born outside Italy, the majority from High Migratory Pressure Countries (HMPC) (74.3%). Incidence rates were significantly lower in subjects born in HMPC in both sexes. Immigrants, in particular born in Asia and Africa, showed lower rates of all site cancer incidence. The lowest IRR for colorectal cancer was observed in males from South-Central America (IRR 0.19, 95%CI 0.09-0.44) and in females from Asia (IRR 0.32, 95%CI 0.18-0.70). The IRR of breast cancer appeared significantly lower than Italian natives in all female populations, except for those coming from HDC. Females from Eastern Europe showed a higher IRR for cervical cancer (IRR 2.02, 95%CI 1.57-2.61).

**Conclusion:**

Cancer incidence was found lower in subjects born outside Italy, with differences in incidence patterns depending on geographical area of origin and the cancer type in question. Further studies, focused on the country of birth of the immigrant population, would help to identify specific risk factors influencing cancer incidence.

## Introduction

In Italy, there are more than 5 million immigrant residents, who represent 8.6% of the total population (1). Among the regions with the highest number of immigrants, Veneto (north-eastern Italy, 4,838,253 inhabitants as of January 1, 2023) is placed in the fourth position, with almost 493,000 migrant people, accounting for more than 10% of the resident population (2).

Immigrants represent a vulnerable population, due to their socioeconomic condition, which affects their engagement in preventive initiatives, disease identification, and healthcare access (3). Poor disease management, lack of check-ups and screening test, delayed diagnosis and inadequate treatments were found to be more frequent in migrants (4, 5).

Variations in risk factors for cancer between immigrants and native people are significant - as well as among immigrants coming from different countries- who show a lower incidence rate for some cancer types in respect of non-immigrants, and vice versa (6–8). In general, native populations show higher cancer rates than immigrants, with some exceptions. Furthermore, the prevalence of risk factors seems to be influenced by the length of stay and age distribution of immigrants (9).

Few studies have been conducted on cancer incidence in immigrants in Italy (10–13). Most studies focused on the prevalence of cervical human papillomavirus infection and related cancer (12, 13). A systematic review of the literature, including studies published between 1996 and 2016, provided a scattered coverage of epidemiological indexes in local settings (14). This review confirms how cancer incidence is generally lower for immigrants than for natives; however, cancers with an infective etiology may have, among immigrants coming from some countries, a great relevance.

Cancer registries collect data on new cancer diagnoses in the population residing in the area covered by the registry. Country of birth is one of the variables collected, and it is well known how being born in a specific geographical area may influence cancer incidence, since it is correlated both with different lifestyle habits (lifestyle, nutrition, reproductive history, etc.) and with a different prevalence of viral and bacterial infections and can be used as a proxy for these factors (15–17).

The aim of our study was to investigate whether there are differences in cancer incidence by geographical area of origin in subjects residing in the Veneto Region, focusing on the cancer sites with the highest occurrence in the immigrant population.

## Materials and methods

### The Veneto tumour registry

The Veneto Tumour Registry (VTR) is a regional, population-based cancer registry, formally established in 2010, with complete regional coverage (4.9 million inhabitants) since 2014. It collects all cancer cases occurred in subjects resident in the Veneto region, including also those cases diagnosed outside the region.

The information for the registry is extracted from the medical records and includes demographics, data on tumour site and morphological classification. The place of birth or, for foreign-born patients, the country of birth is also registered.

### Study population

We selected all incident cancer cases recorded during the years 2015-2019 in residents of the Veneto Region, including only cases occurred in subject aged 20+ years. For each person resident in Veneto (including cancer cases), the information on the country of birth was retrieved from the Regional Health Service’s population lists through the “cadastral code” contained in positions 12-15 of the fiscal code. For Italian citizens born abroad, the foreign country of birth is considered: in this case, the four-digits code begins with the letter “Z” followed by the identification number of the country.

Based on the country of birth, subjects were classified into six geographical areas of origin:

- born in Italy;
- born in one of the 40 Highly Developed Countries (HDC): 15 countries of the European Union (Austria, Belgium, Cyprus, Denmark, Finland, France, Germany, Greece, Ireland, Luxembourg, Malta, Netherlands, Portugal, Spain, Sweden), 15 other European countries (Andorra, Faroe Islands, Gibraltar, Guernsey, Iceland, Isle of Man, Jersey, Liechtenstein, Monaco, Norway, San Marino, Sark, Switzerland, United Kingdom, Vatican City), 5 North American countries (Bermuda, Canada, Greenland, Saint Pierre and Miquelon, United States of America), 2 countries in Oceania (Australia and New Zealand), 3 countries in Asia (Israel, South Korea and Japan);
- born in one of the 22 Eastern European countries (Albania, Belarus, Bosnia-Herzegovina, Bulgaria, Croatia, Czech Republic, Estonia, Hungary, Kosovo, Latvia, Lithuania, Moldova, Montenegro, North Macedonia, Poland, Romania, Russian Federation, Serbia, Slovakia, Slovenia, Turkey, Ukraine);
- born in one of the remaining 62 countries in Asia or Oceania (Asia)
- born in one of the 56 African countries (Africa)
- born in one of the 44 countries of Central and South America

Countries included in the groups Eastern European countries, Asia, Africa and Central and South America were classified as High Migratory Pressure Countries (HMPC)

A complete list of the countries included in each of the geographical areas of origin is provided in **Supplementary Table S1** in the **Appendix**.

### Statistical analysis

To calculate the denominator of incidence rates, we used Regional Health Service’s population lists, stratified by age, sex, calendar year and geographical area of origin.

Age-standardized incidence rates (ASR), and relative 95% confidence intervals, were calculated, based on the 2013 European standard population, for all cancer sites, for three groups (Italy, HDC, HMPC), according to the country of birth, and sex and sex. We also calculated ASR for the ten more common cancer sites in the immigrant population.

Incidence rate ratio (IRR), and relative 95%CI, with Italy as reference, were also calculated, for all cancer sites and for colorectal, liver, breast and cervical cancer separately, stratifying for geographical area of origin and sex, where applicable.

All statistical analyses were performed using SEER*Stat, 8.4.2 version (2023) and R software, version 4.2.1 (2022).

## Results

Our study population is represented by more than 3.4 million people born in Italy, and 532,112 subjects born abroad aged 20+ years and resident in the Veneto Region (**Table 1**). Almost half of the subjects born abroad (47.8%) come from Eastern Europe, 18.7% from Africa and 14.3% from Asian countries, 7.5% from South-central America and 11.6% from highly developed countries (HDC). The median age is lower for all immigrants, regardless of geographical areas of origin, as compared to natives. Most of the subjects born abroad are included in the age class 20-39 years (43%). In the oldest age class, people coming from High Migratory Pressure Countries (HMPC) accounted only for 1.4% of all study population. The percentage of male subjects varies by geographical area, ranging from 35.6% in people coming from South - Central America to 57.5% in people coming from African countries.

**Table 1:** Characteristics of the study population, by geographical area of origin.

Geographical area of origin	Mid Population 2015-2019	Median age (years)	Male		20 - 39 years		40 - 59 years		60 - 79 years		80+ years	
			n.	%	n.	%	n.	%	n.	%	n.	%
Italy	3,460,511	53	1,682,822	48.6	813,487	23.5	1,316,515	38.0	1,008,972	29.2	321,537	9.3
HDC	61,655	52	26,206	42.5	8,712	14.1	38,357	62.2	11,733	19.0	2,853	4.6
HMPC	470,457	40	214,171	45.5	221,996	47.2	202,057	42.9	42,073	8.9	4,331	0.9
Africa	99,751	41	57,346	57.5	44,004	44.1	45,475	45.6	9,217	9.2	1,055	1.1
Asia	76,278	38	41,359	54.2	41,463	54.4	30,801	40.4	3,838	5.0	176	0.2
South-central America	39,817	42	14,170	35.6	17,449	43.8	17,062	42.9	4,767	12.0	539	1.4
Eastern Europe	254,611	41	101,296	39.8	119,080	46.8	108,719	42.7	24,251	9.5	2,561	1.0

HDC, Highly Developed Countries.

HMPC, High Migratory Pressure Countries.

In the years 2015-2019, we recorded 159,486 cancer cases (excluding non-melanoma skin cancer) in subjects aged more than 20 years old; 5.2% cases occurred in foreign-born people, in particular from countries with high migration (HMPC) (74.3%) (**Table 2**).

**Table 2:** Number of cancer cases and age standardized (European standard population 2013) incidence rate (ASR) (x 100,000), by sex and geographical area of origin for subjects aged 20+ years.

Geographical area of origin	Male				Female			
	n.	ASR	95%CI		n.	ASR	95%CI	
Italy	80,423	878.5	872.4	884.7	70,848	661.2	656.2	666.2
HDC	864	885.0	812.7	961.8	1,246	647.8	608.7	689.0
HMPC	2,374	647.2	612.5	683.2	3,731	483.2	462.9	504.1
Africa	643	598.5	539.8	661.4	513	399.4	358.2	443.7
Asia	257	498.0	367.6	653.2	330	441.3	342.3	557.2
South-central America	228	760.8	621.1	919.3	432	458.3	403.8	517.8
Eastern Europe	1,246	691.7	642.8	742.9	2,456	534.5	506.2	563.8

HDC, Highly Developed Countries.

HMPC, High Migratory Pressure Countries.

All sites but skin non-melanoma.

Differences were reported in the sex distribution of cancer cases, with a higher percentage of cases occurring in females born abroad in respect of Italian natives (60.6% vs 46.8% respectively). People coming from HDC showed rates similar to those of Italian natives, both in males and females. Incidence rates were significantly lower in subject born in HMPC in both sexes, in particular in subjects coming from Asian and African countries. Incidence rates were significantly lower in females from South-Central American, but not in males. Among HMPC, the highest rates were observed in subjects coming from Eastern European countries in both sexes.

**Figure 1:**
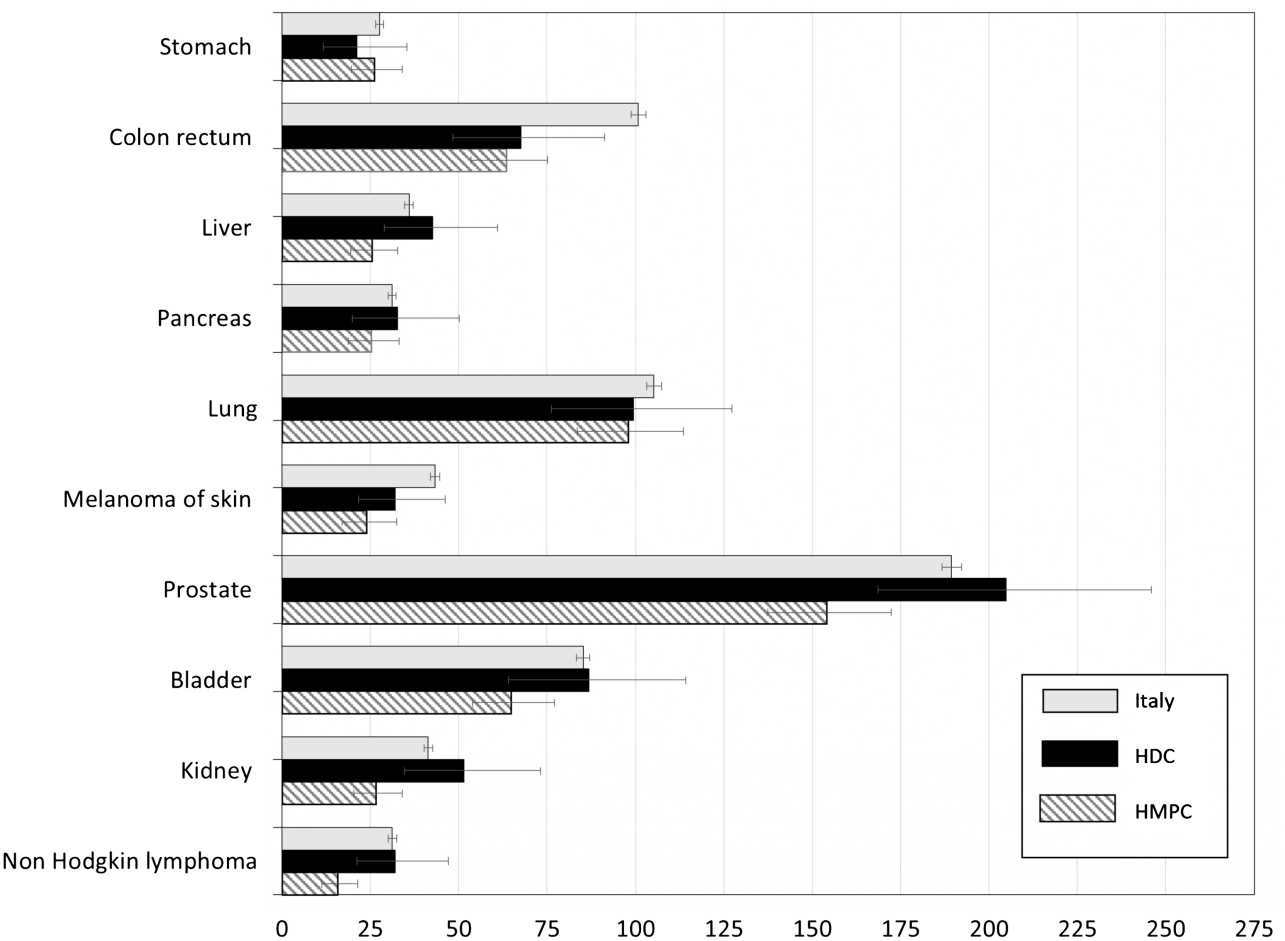
Age-standardized incidence rates (ASR) per 100,000 and relative 95% CI by cancer site and geographical area of origin for subjects aged 20+ years. Males. HDC, Highly Developed Countries; HMPC, High Migratory Pressure Countries.

**Figure 1** reports Age Standardised Rates (ASR) (x 100,000) and relative 95% Confidence Intervals (CI) for male subjects by the most common cancer sites. No statistically significant differences in incidence emerged between males born in Italy and in HDC, while males from HMPC showed rates significantly lower than Italian natives for all cancer sites investigated, but stomach, lung and pancreas. Prostate represented the cancer site with the highest incidence rate (>150 per 100,000), regardless of country of birth. Incidence rates of lung cancer reached almost 100 per 100,000 in HDC and HMPC (99.4 and 97.9 respectively). Statistically significant differences emerged for colorectal cancer, with males born in Italy showing a rate of 100 per 100,000, while in subjects coming from HMPC and HDC we observed a rate of 63.6 and 67.5 per 100,000 respectively. Males from HMPC showed significantly lower incidence rates of bladder cancer when compared with Italian natives (64.8 vs 85.2).

**Figure 2:**
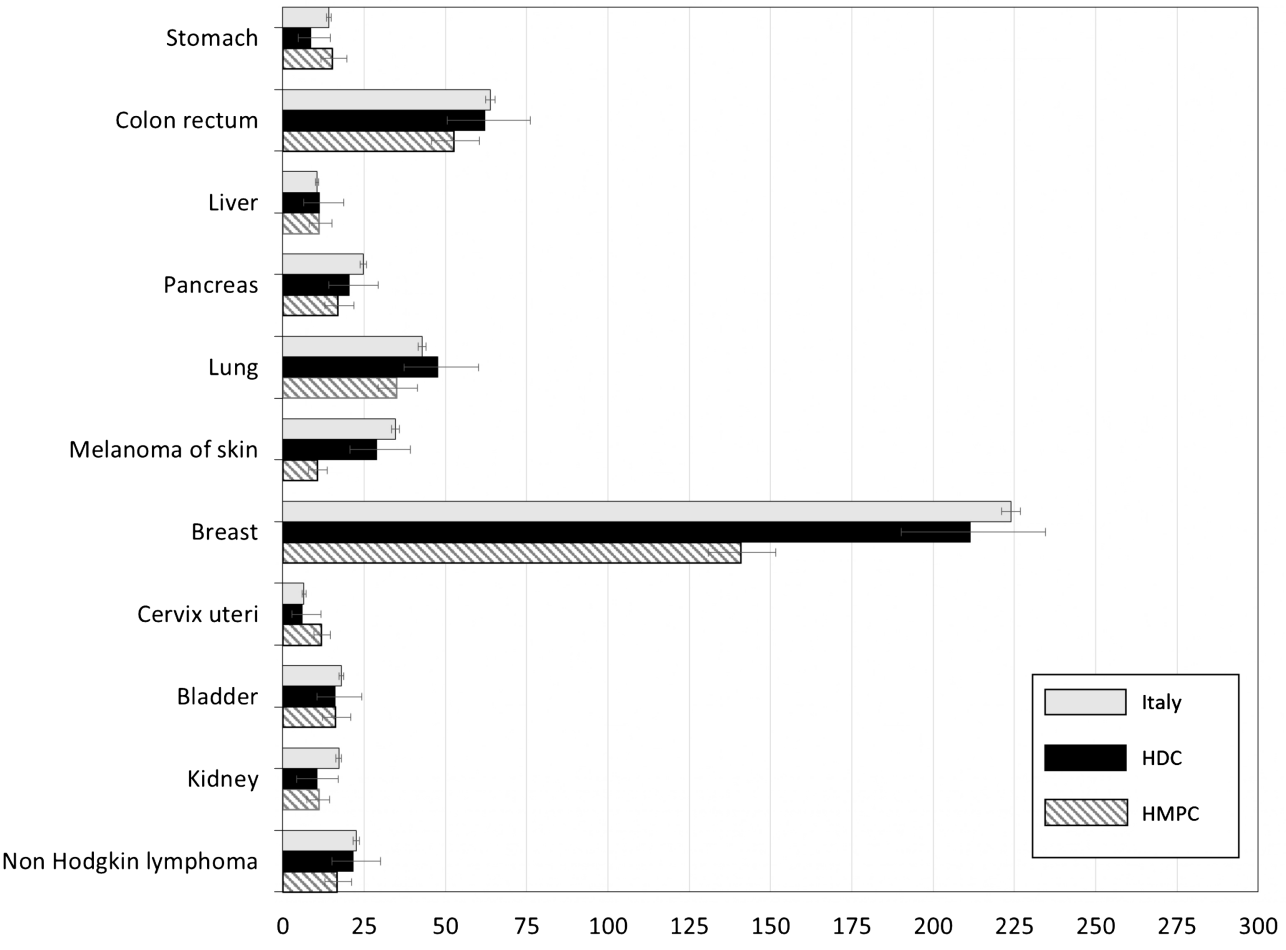
Age-standardized incidence rates (ASR) per 100,000 and relative 95% CI by cancer site and geographical area of origin for subjects aged 20+ years. Females. HDC, Highly Developed Countries; HMPC, High Migratory Pressure Countries.

In **Figure 2** Age Standardised Rates (ASR) for female subjects by the most common cancer sites were reported. Also for the female population, statistically significant differences between those born Italy and in HDC were not observed. Breast cancer represented the cancer site with the highest incidence rate, regardless of country of birth, with females from HMPC showed a significantly lower rate when compared with Italian natives (140.9 per 100,000 vs 224 per 100,000). Differences in colorectal cancer were less marked than those in males, but still significant, with a rate in females from HMPC of 52.7 per 100,000 vs 63.7 per 100,000 in those born in Italy.

Cutaneous melanoma was more frequent in females from Italy (34.6 per 100,000 vs 10.5 per 100,000 in those born in HMPC). Significantly lower rates, when compared with Italian natives, emerged in females from HMPC also for pancreas, lung, kidney and Non Hodgkin Lymphoma. Cervical cancer incidence was almost doubled in females born in HMPC (11.8 per 100,000 vs 6.5 per 100,000 in Italian natives). All ASR data by sex were reported in **Supplementary Tables S2** and **S3** in the appendix.

**Figure 3:**
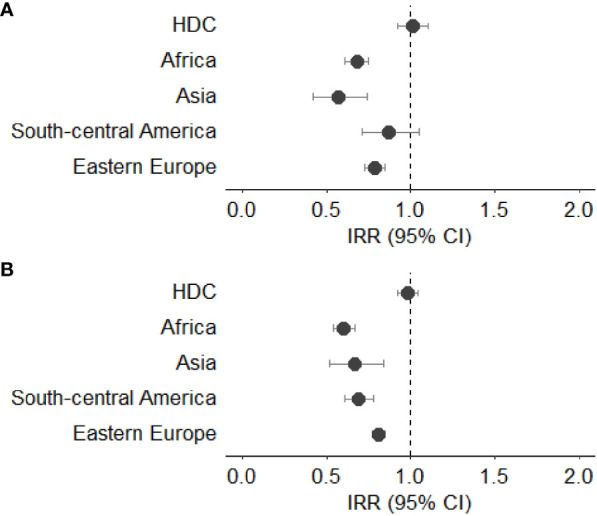
**(A, B)** Incidence rate ratio (IRR) with 95% CI for all cancers (excluding non-melanoma skin cancer) in males **(A)** and females **(B)**. HDC, Highly Developed Countries.

**Figure 4:**
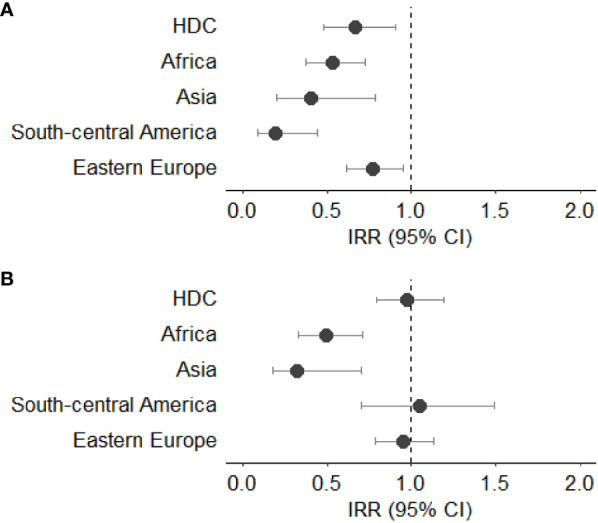
**(A, B)** Incidence rate ratio (IRR) with 95% CI for colorectal cancer in males **(A)** and females **(B)**. HDC, Highly Developed Countries.

**Figure 5:**
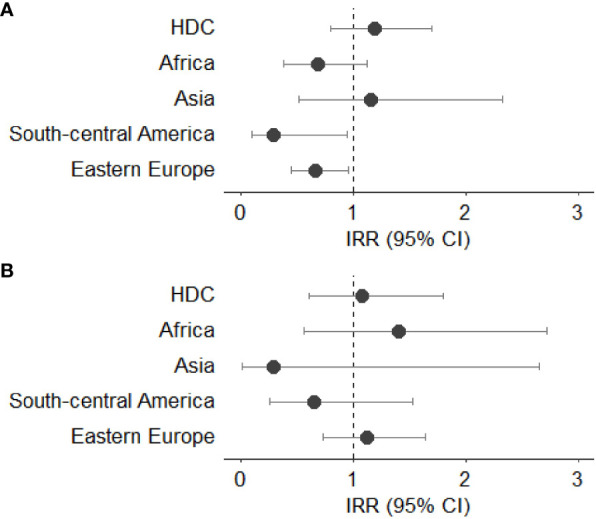
**(A, B)** Incidence rate ratio (IRR) with 95% CI for liver cancer in males **(A)** and females **(B)**. HDC, Highly Developed Countries.

**Figure 6:**
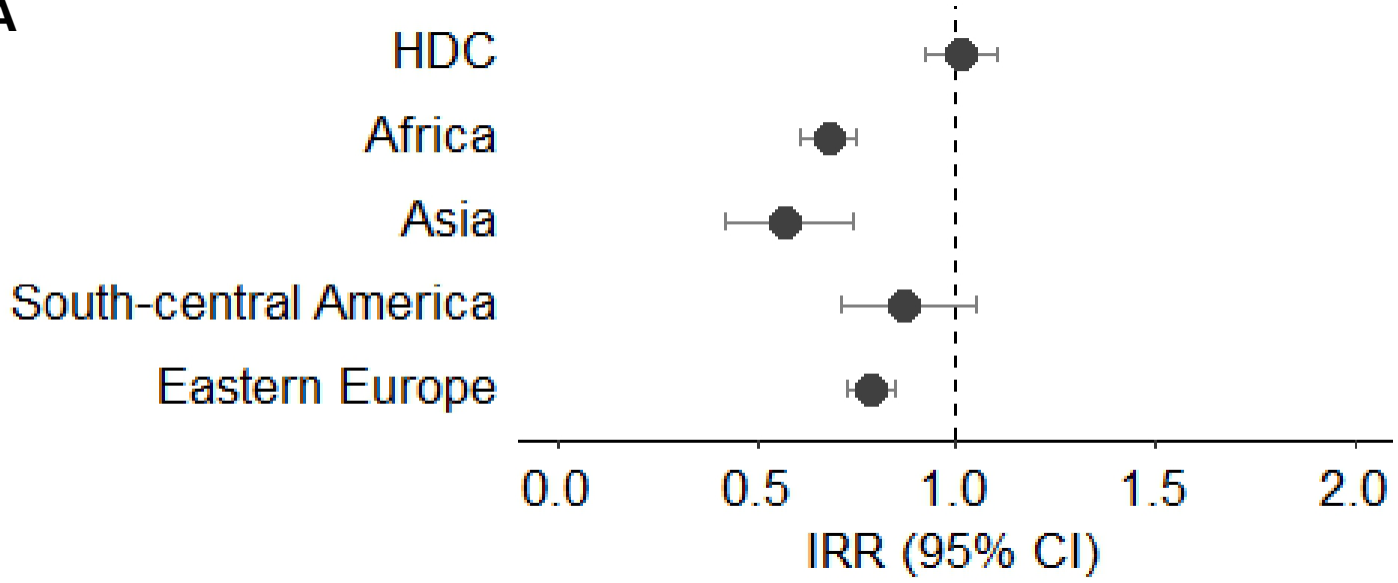
Incidence rate ratio (IRR) with 95% CI for female breast cancer. HDC, Highly Developed Countries.

**Figure 7:**
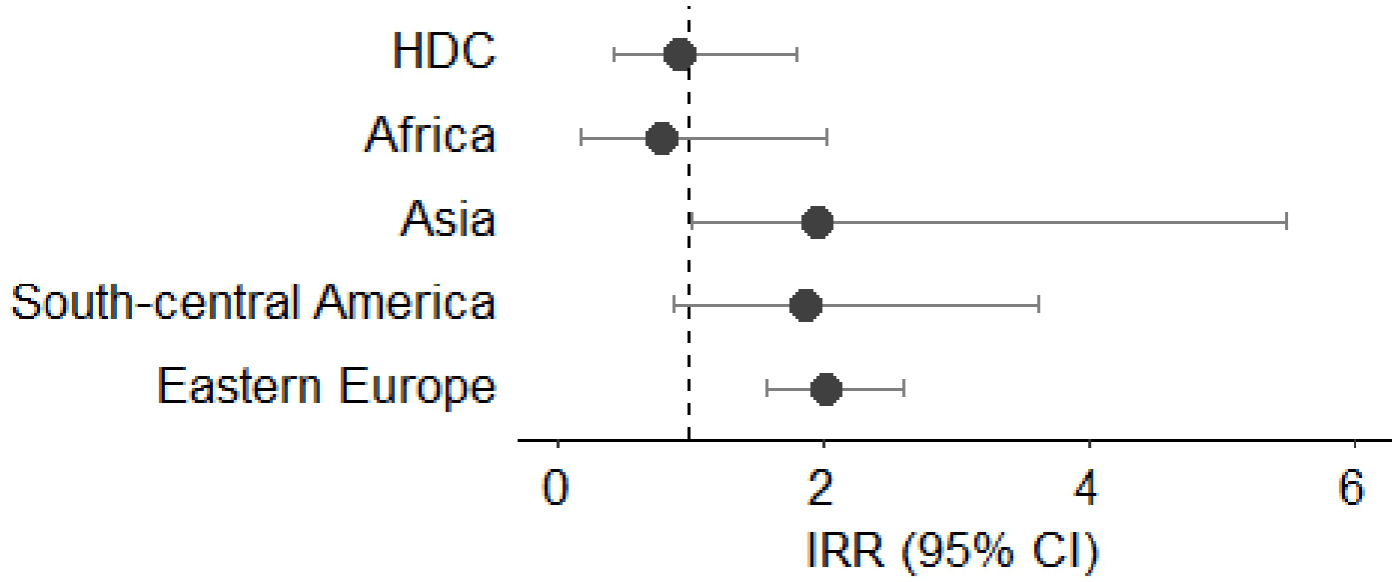
Incidence rate ratio (IRR) with 95% CI for cervical cancer. HDC, Highly Developed Countries.

**Figures 3**–**7** show Incidence Rate Ratios (IRRs) with 95% Confidence intervals, for the five main geographical areas compared with Italy (reference = 1).

Compared with Italian natives, male immigrants showed lower rates of cancer incidence, with a statistically significant reduction in those coming from Asia (IRR 0.57, 95%CI 0.42-0.74), Africa (IRR 0.68, 95%CI 0.61-0.75), and Eastern Europe (IRR 0.79, 95%CI 0.73-0.85). Females born in Africa showed a lower incidence rate than Italians (IRR 0.60, 95%CI 0.54-0.67), as well as those born in Asia (IRR 0.67, 95%CI 0.52-0.84) and in South – Central America (IRR 0.69, 95%CI 0.61-0.78). No significant differences were observed for subjects from HDC when compared with Italian natives in both sexes (**Figures 3A, B**).

The IRRs for colorectal cancer were lower for all male subjects born outside Italy when compared to Italian natives. In particular, the lowest IRR was observed in those coming from South - Central America (IRR 0.19, 95%CI 0.09-0.44), followed by those from Asia (IRR 0.41, 95%CI 0.20-0.79) and Africa (IRR 0.53, 95%CI 0.38-0.73). Differently from male subjects, only females from Asia and Africa showed lower IRR for colorectal cancer (IRR 0.32, 95%CI 0.18-0.70 and IRR 0.50, 95%CI 0.33-0.71, respectively). No statistically significant differences were observed in females born in Eastern Europe, HDC and South - Central America (**Figures 4A, B**).

Only males from South-Central America and Eastern Europe reported significantly lower incidence rate ratio of liver cancer (IRR 0.29, 95%CI 0.10-0.94 and IRR 0.67, 95%CI 0.45-0.9, respectively). No statistically significant differences were observed in the incidence of liver cancer in female subjects born outside Italy. (**Figures 5A, B**).

The incidence rate ratio of breast cancer appeared significantly lower than Italian natives in all female immigrant population, except for those coming from HDC. The IRR was particularly low in females from Asian and African countries (IRR 0.56, 95%CI 0.40-0.79 and IRR 0.60, 95%CI 0.50-0.72, respectively) (**Figure 6**).

The IRR for cervical cancer was higher than Italian natives for females from Eastern Europe (IRR 2.02, 95%CI 1.57-2.61) and from Asian countries (IRR 1.97, 95%CI 1.00-5.49) (**Figure 7**).

## Discussion

This is the largest Italian study, to our knowledge, investigating cancer incidence in immigrants, stratified by geographical area of origin. We found that subjects born in countries with high migration (HMPC) showed lower standardized incidence rates compared with Italian natives for all cancer sites considered, but cervical cancer. Moreover, incidence rate ratios (IRR) showed lower rates of cancer incidence in both male and female subjects born abroad, regardless of the country of birth. Our results are in line with other studies conducted in Europe: in Norway, Hjerkin et al. (6) reported higher ASRs in Norwegians in respect of all immigrants combined, in both sexes.

Regarding colorectal cancer incidence rates, we observed lower rates for foreign-born males of all geographical areas, in particular from South Central America and Asia, similarly to other studies conducted in Italy and Canada (10, 18). Sex differences emerged for this cancer site, since we found that, differently from males, females born in Eastern Europe and South Central America showed incidence rates similar to those of Italian natives.

Differences in colorectal cancer incidence may be related to the adherence to screening programs, which in Italy cover the majority of the resident population, inviting all the resident population without any difference as regards citizenship (19). A study conducted in Italy by Turrin et al. found that compliance with invitation to screening in immigrants was reduced by one-third (34% vs. 51%); immigrants showed also a lower prevalence of colorectal cancer at the first screening test (20).

We observed a reduced risk of liver cancer in foreign-born men, especially those coming from South Central America and Eastern Europe, but not in women. These results are in line with another study conducted in Italy (10). Chronic infection with hepatitis B virus (HBV) or hepatitis C virus (HCV), alcohol abuse, and obesity are all risk factors associated with liver cancer, and they may be differently present in the foreign-born population, as well as according to sex. This could partly explain the differences encountered. In addition, a different presence of metabolic conditions and fat-related liver disease, which have become increasingly prominent risk factors, could have played a role (21, 22).

We reported lower breast cancer incidence rates in all female foreign-born populations, except for women born in HDC, when compared with Italian natives. Reasons for lower breast cancer risks in women born abroad compared to native can partly be explained by differences in reproductive and lifestyle patterns (23). More specifically, reproductive indicators, such as the early age at menarche and higher age at first birth, as well as the number of children, breastfeeding behaviours and the use of hormonal therapies in postmenopausal women represent key risk factors in the carcinogenesis of breast cancer (24). Migrant women from less developed countries often exhibit many protective risk factors that reduce their breast cancer risk (25). Low breast cancer incidence in migrant women of non-Western origin have also been reported by several studies conducted in other European countries (26, 27).

We observed a higher risk of cervical cancer among women from Eastern Europe, which might be explained by the high prevalence of HPV infection in that region (28). This finding is in line with other Italian studies (10, 11). In Italy was also reported that 58% Eastern European vs. 19% of Italy-born women are HPV-positive (29). The higher risk of cervical cancer is also influenced by HPV screening participation, which in Italy varies among immigrants from different geographical areas. A study conducted by Battagello et al. (30) found that Eastern European women showed lower attendance, when compared with Italian natives (45.5% vs 56.4%).

In general, our study confirms findings of other recent studies of immigrants from low-income to high-income countries. Differences in cancer rates, overall and for specific cancer types, are likely due to a combination of different factors, such as differences in lifestyle and socioeconomic factors, reason for immigration (i.e. war/conflicts, economic causes), and the “healthy migrant effect” (6).

Certain lifestyle factors associated with cancer risk may differ between immigrants and host populations. In particular, diet may be healthier and the prevalence of obesity and alcohol consumption lower in immigrants than in some high-income host populations before and immediately after arrival (31–33).

Studies on migrant populations have shown that with migration the oncological risk typical of the area of origin is also transferred. This is, in general in first-generation migrants, similar to that of the population of origin. In subjects born abroad and arrived at very young age in Italy, the oncological risk may become similar of that of the host country, as a result of a rapid integration and thus the acquisition of environmental factors typical of the country of immigration. After moving to the host country, in fact, immigrants go through various degrees of acculturation. Many factors, for example, socioeconomic status and occupation, can influence the acculturation process, and affect cancer incidence (34).

Immigrants from low- income countries are likely to have lower socioeconomic status than Italian natives have, and they are over-represented in occupations where they could be exposed to risk factors associated with cancer (35). In addition, widespread high-income diets and increasing obesity prevalence worldwide could influence acculturation in recent immigrants (6).

A strength of this study is the use of a cancer registry with a high validity to identify accurately all incident cases occurred in our study population. Secondly, the study design enabled us to identify correctly all foreign-born in Italy, stratifying for main geographical areas of origin, adding, thus, valuable data to the current literature on cancer epidemiology in migrants.

This study has, also, some limits. The “salmon bias” hypothesis would suggest that as immigrants age or fall ill they move back to die to their country of origin (36); however, this should not have an impact in our study, since health services and treatments are most likely better in Italy, and many immigrants have already been joined by their families. Furthermore, in Italy the immigrant population is still relatively young, especially immigrants from low-income countries, and may have not yet reached the age groups characterized with high lung, colorectal and prostate cancer rates. This is especially the case for prostate cancer, where 85% of cases are diagnosed in subjects older than 65 years, after which risk increase with age (37). Nonetheless, the rates presented in our study are age-standardized, that is, adjusted for the effect of differences in age distribution across populations. Lastly, although we used data from a regional registry, which covers the 8% of the Italian population, our study gives a reliable picture of cancer incidence in the north of Italy. A recent study (38), based on 36 local and regional cancer registries, reported a north - south gradient in all cancers incidence rates, in both male and female patients, with regions of the north of Italy showing higher rates similar to those reported in our study.

## Conclusion

Our study showed that the total cancer incidence was lower in foreign- born subjects than in Italian natives, with differences in incidence patterns depending on geographical area of origin and the cancer type in question. The extent of such differences varied according to sex and geographical area of origin. Subjects from highly developed countries had a relatively similar cancer burden compared to Italian-born, with high incidence of lifestyle-related cancers. Further studies, focusing on the country of birth of the immigrant population, would help to better identify specific risk factors influencing cancer incidence.

## Data availability statement

The datasets generated and/or analyzed during the current study are not publicly available because of privacy reasons. Requests to access the datasets should be directed to eliana.ferroni@azero.veneto.it.

## Ethics statement

The Italian legislation identifies regional and national health authorities as collectors of personal data for surveillance purposes without explicit individual consent. The approval of a research ethic committee is not required, because this study is a descriptive analysis of anonymous aggregate data without any direct or indirect intervention on patients (Decreto del Presidente del Consiglio dei Ministri, 3/3/2017, Identificazione dei sistemi di sorveglianza e dei registri di mortalita`, di tumori e di altre patologie, 17A03142, GU Serie Generale n.109 del 12-05-2017). Available at: www.gazzettaufficiale.it/eli/id/2017/05/12/17A03142/sg (last accessed April 22, 2024).

## Author contributions

EF: Conceptualization, Methodology, Validation, Writing – original draft, Writing – review & editing. SG: Data curation, Formal analysis, Methodology, Validation, Writing – review & editing. AA: Data curation, Writing – review & editing. SB: Data curation, Writing – review & editing. MB: Data curation, Writing – review & editing. EB: Data curation, Writing – review & editing. EC: Data curation, Writing – review & editing. AD: Data curation, Writing – review & editing. AG: Funding acquisition, Resources, Writing – review & editing. AF: Data curation, Writing – review & editing. LM: Data curation, Writing – review & editing. DM: Data curation, Writing – review & editing. SR: Data curation, Writing – review & editing. JS: Funding acquisition, Resources, Writing – review & editing. CS: Data curation, Writing – review & editing. SZ: Writing – review & editing, Data curation. MZ: Conceptualization, Methodology, Supervision, Validation, Writing – original draft, Writing – review & editing.
