# Supplementary material for: Cancer incidence in immigrants by geographical area of origin: data from the Veneto Tumour Registry, Northeastern Italy
Source: Front Oncol. 2024 May 28;14:1372271. doi: 10.3389/fonc.2024.1372271 (PMC11165053; doi:10.3389/fonc.2024.1372271)
Supplement: Supplementary file 1 [file Table_1.docx]

**Appendix**

Table 1S. Countries included in the geographical area of origin.

| High Migratory Pressure Countries (HMPC): Eastern Europe | Albania, Belarus, Bosnia-Herzegovina, Bulgaria, Croatia, Czech Republic, Estonia, Hungary, Kosovo, Latvia, Lithuania, Moldova, Montenegro, North Macedonia, Poland, Romania, Russian Federation, Serbia, Slovakia, Slovenia, Turkey, Ukraine |
| --- | --- |
| High Migratory Pressure Countries (HMPC): Africa | Algeria, Angola, Benin, Botswana, Burkina Faso, Burundi, Cameroon, Cabo Verde, Central African Republic, Chad, Comoros, Congo, Côte d’Ivoire, Democratic Republic of the Congo, Djibouti, Egypt, Equatorial Guinea, Eritrea, Eswatini, Ethiopia, Gabon, Gambia, Ghana, Guinea, Guinea-Bissau, Kenya, Lesotho, Liberia, Libya, Madagascar, Malawi, Mali, Mauritania, Mauritius, Morocco, Mozambique, Namibia, Niger, Nigeria, Rwanda, Saint Helena, Sao Tomé and Principe, Senegal, Seychelles, Sierra Leone, Somalia, South Africa, South Sudan, Sudan, Togo, Tunisia, Uganda, United Republic of Tanzania, Western Sahara, Zambia,  Zimbabwe |
| High Migratory Pressure Countries (HMPC): Asia | Afghanistan, Armenia, Azerbaijan, Bahrain, Bangladesh, Bhutan, Brunei Darussalam, Cambodia, China, Cook Islands, Democratic People's Republic of Korea, Fiji, French Polynesia, Georgia, India, Indonesia, Iran (Islamic Republic of), Iraq, Jordan, Kazakhstan, Kiribati, Kuwait, Kyrgyzstan, Lao People's Democratic Republic, Lebanon, Malaysia, Maldives, Marshall Islands, Micronesia (Federated States of), Mongolia, Myanmar, Nauru, Nepal, New Caledonia, Oman, Pakistan, Palau, State of Palestine, Papua New Guinea, Philippines, Pitcairn, Qatar, Samoa, Saudi Arabia, Singapore, Solomon Islands, Sri Lanka, Syrian Arab Republic, Taiwan, Tajikistan, Thailand, Terres australes et antarctiques françaises, Timor- Leste, Tonga, Turkmenistan, Tuvalu, United Arab Emirates, Uzbekistan, Vanuatu, Viet Nam, Wallis and Futuna, Yemen |
| High Migratory Pressure Countries (HMPC): South-central America | Anguilla, Antigua and Barbuda, Argentina, Aruba, Bahamas, Barbados, Belize, Bolivia, Brazil, British Virgin Islands, Cayman Islands, Chile, Colombia, Costa Rica, Cuba, Curaçao, Dominica, Dominican Republic, Ecuador, El Salvador, Falkland Islands, Grenada, Guatemala, Guyana, Haiti, Honduras, Jamaica, Mexico, Montserrat, Nicaragua, Panama, Paraguay, Peru, Saint-Barthélemy, Saint Kitts and Nevis, Saint Lucia, Saint-Martin (French Part), Saint Vincent and the Grenadines, Sint Maarten (Dutch part), Suriname, Trinidad and Tobago, Turks and Caicos Islands, Uruguay, Venezuela |
| Highly Developed Countries (HDC) | Andorra, Australia, Austria, Belgium, Bermuda, Canada, Cyprus, Denmark, Faroe Islands, Finland, France, Germany, Gibraltar, Greece, Greenland, Guernsey, Iceland, Ireland, Isle of Man, Israel, Japan, Jersey, Liechtenstein, Luxembourg, Malta, Monaco, Netherlands, New Zealand, Norway, Portugal, San Marino, Saint Pierre and Miquelon, Sark, Spain, South Korea, Sweden Switzerland, United Kingdom, United States of America, Vatican City |
