# Supplementary material for: Cancer incidence in immigrants by geographical area of origin: data from the Veneto Tumour Registry, Northeastern Italy
Source: Front Oncol. 2024 May 28;14:1372271. doi: 10.3389/fonc.2024.1372271 (PMC11165053; doi:10.3389/fonc.2024.1372271)
Supplement: Supplementary file 2 [file Table_2.docx]

Table 2S. Age-standardized incidence rates (ASR) per 100,000 and relative 95% CI by cancer site and geographical area of origin for subjects aged 20+ years. Males

| **Site** | **Country of birth** | **Rate** | **95% CI** | |
| --- | --- | --- | --- | --- |
| Non Hodgkin lymphoma | HMPC | 15.7 | 11.2 | 21.4 |
|  | HDC | 32 | 21.1 | 47.1 |
|  | Italy | 31.2 | 30.1 | 32.4 |
|  |  |  |  |  |
| Kidney | HMPC | 26.6 | 20.3 | 34.0 |
|  | HDC | 51.4 | 34.7 | 73.1 |
|  | Italy | 41.4 | 40.1 | 42.7 |
|  |  |  |  |  |
| Bladder | HMPC | 64.8 | 53.8 | 77.1 |
|  | HDC | 86.8 | 64.1 | 114.3 |
|  | Italy | 85.2 | 83.3 | 87.1 |
|  |  |  |  |  |
| Prostate | HMPC | 154.2 | 137.4 | 172.3 |
|  | HDC | 204.8 | 168.6 | 245.9 |
|  | Italy | 189.4 | 186.6 | 192.2 |
|  |  |  |  |  |
| Melanoma of skin | HMPC | 23.9 | 16.9 | 32.5 |
|  | HDC | 32 | 21.7 | 46.2 |
|  | Italy | 43.2 | 41.9 | 44.6 |
|  |  |  |  |  |
| Lung | HMPC | 97.9 | 83.6 | 113.6 |
|  | HDC | 99.4 | 76.2 | 127.3 |
|  | Italy | 105.2 | 103.1 | 107.3 |
|  |  |  |  |  |
| Pancreas | HMPC | 25.2 | 18.7 | 33.1 |
|  | HDC | 32.6 | 19.8 | 50.2 |
|  | Italy | 31.2 | 30 | 32.3 |
|  |  |  |  |  |
| Liver | HMPC | 25.4 | 19.4 | 32.6 |
|  | HDC | 42.7 | 29 | 60.9 |
|  | Italy | 35.9 | 34.7 | 37.2 |
|  |  |  |  |  |
| Colon rectum | HMPC | 63.6 | 53.4 | 75.1 |
|  | HDC | 67.5 | 48.4 | 91.2 |
|  | Italy | 100.8 | 98.8 | 102.9 |
|  |  |  |  |  |
| Stomach | HMPC | 26.2 | 19.6 | 34 |
|  | HDC | 21.2 | 11.7 | 35.4 |
|  | Italy | 27.7 | 26.6 | 28.8 |

HDC, Highly Developed Countries.

HMPC, High Migratory Pressure Countries.
