# Supplementary material for: Cancer incidence in immigrants by geographical area of origin: data from the Veneto Tumour Registry, Northeastern Italy
Source: Front Oncol. 2024 May 28;14:1372271. doi: 10.3389/fonc.2024.1372271 (PMC11165053; doi:10.3389/fonc.2024.1372271)
Supplement: Supplementary file 3 [file Table_3.docx]

Table 3S Age-standardized incidence rates (ASR) per 100,000 and relative 95% CI by cancer site and geographical area of origin for subjects aged 20+ years. Females

| **Site** | **Country of birth** | **Rate** | **95% CI** | |
| --- | --- | --- | --- | --- |
| Non Hodgkin lymphoma | HMPC | 16.6 | 12.8 | 21.1 |
|  | HDC | 21.5 | 15.2 | 30.0 |
|  | Italy | 22.6 | 21.7 | 23.6 |
|  |  |  |  |  |
| Kidney | HMPC | 11.1 | 8.3 | 14.4 |
|  | HDC | 10.6 | 6.5 | 17.0 |
|  | Italy | 17.2 | 16.4 | 18.0 |
|  |  |  |  |  |
| Bladder | HMPC | 16.1 | 12 | 20.8 |
|  | HDC | 16.1 | 10 | 24.2 |
|  | Italy | 18.0 | 17 | 18.8 |
|  |  |  |  |  |
| Cervix uteri | HMPC | 11.8 | 9.6 | 14.5 |
|  | HDC | 6 | 2.9 | 11.7 |
|  | Italy | 6.5 | 6 | 7.1 |
|  |  |  |  |  |
| Breast | HMPC | 140.9 | 130.8 | 151.6 |
|  | HDC | 211.5 | 190.3 | 234.6 |
|  | Italy | 224 | 221 | 226.9 |
|  |  |  |  |  |
| Melanoma of skin | HMPC | 10.5 | 8 | 13.7 |
|  | HDC | 28.8 | 21 | 39.3 |
|  | Italy | 34.6 | 33 | 36 |
|  |  |  |  |  |
| Lung | HMPC | 35.1 | 29 | 41 |
|  | HDC | 47.6 | 37 | 60 |
|  | Italy | 42.7 | 42 | 44 |
|  |  |  |  |  |
| Pancreas | HMPC | 17.0 | 13 | 21.8 |
|  | HDC | 20.5 | 14 | 29.4 |
|  | Italy | 24.7 | 23.8 | 25.7 |
|  |  |  |  |  |
| Liver | HMPC | 11.2 | 8 | 15.2 |
|  | HDC | 11.3 | 6.4 | 18.7 |
|  | Italy | 10.5 | 9.9 | 11.1 |
|  |  |  |  |  |
| Colon rectum | HMPC | 52.7 | 45.6 | 60.5 |
|  | HDC | 62.2 | 50.5 | 76 |
|  | Italy | 63.7 | 62.3 | 65.3 |
|  |  |  |  |  |
| Stomach | HMPC | 15.3 | 11.6 | 19.6 |
|  | HDC | 8.6 | 4.8 | 14.7 |
|  | Italy | 14.1 | 13.5 | 14.9 |

HDC, Highly Developed Countries.

HMPC, High Migratory Pressure Countries.
